# The Effect of a Physical Activity Coaching Intervention on Accelerometer-Measured Sedentary Behaviours in Insufficiently Physically Active Ambulatory Hospital Patients

**DOI:** 10.3390/ijerph18115543

**Published:** 2021-05-22

**Authors:** Stephen Barrett, Stephen Begg, Paul O′Halloran, Michael Kingsley

**Affiliations:** 1 La Trobe Rural Health School, La Trobe University, P.O. Box 199, Bendigo, Victoria 3552, Australia; Sbarrett@bendigohealth.org.au (S.B.); s.begg@latrobe.edu.au (S.B.); 2 Bendigo Health Care Group, Health Promotion Department, P.O. Box 126, Bendigo, Victoria 3552, Australia; 3 School of Psychology and Public Health, La Trobe University, Bundoora, Victoria 3086, Australia; p.ohalloran@latrobe.edu.au; 4 Holsworth Research Initiative, La Trobe Rural Health School, La Trobe University, P.O. Box 199, Bendigo, Victoria 3552, Australia; 5 Department of Exercise Sciences, University of Auckland, Newmarket 1023, New Zealand

**Keywords:** physical activity, sedentary behaviours, secondary prevention, rural health

## Abstract

Little is known about the impact that physical activity (PA) coaching interventions have on sedentary behaviours. The aim of this study was to investigate if a coaching intervention that increases PA coincidentally influences objectively measured sedentary time in insufficiently physically active adults. We recruited 120 insufficiently physically active ambulatory hospital patients and randomized them to either receive a PA coaching intervention designed to increase objectively measured moderate-to-vigorous-intensity PA (MVPA) or be part of a control group. Participants wore an accelerometer for seven days at baseline, post-intervention (three months) and follow-up (nine months). Changes in the average length of sedentary bouts, proportion of time in sedentary behaviours and number of sedentary bouts were evaluated using mixed-model ANOVAs. At baseline, both groups undertook 67 ± 13 sedentary bouts and spent 69% ± 6% of their time in sedentary behaviours. Compared with control, the intervention group decreased the number of sedentary bouts by 24% and the proportion of time in sedentary behaviours by 7% (*p* < 0.001). Significant changes were not observed between the groups for average length of sedentary bouts. The PA intervention led to a decrease in the number of sedentary bouts and proportion of time in sedentary behaviours. Future research should investigate PA coaching interventions designed to target simultaneous changes in MVPA and sedentary behaviours.

## 1. Introduction

Both insufficient physical activity (PA) and sedentary behaviours contribute significantly to ill health and are major challenges to public health [1]. Sedentary behaviour refers to any waking activity, performed in a sitting, lying or reclining position, with an energy expenditure of ≤1.5 metabolic equivalents (METs) [2]. Insufficient PA refers to not meeting PA guidelines of 150 min of moderate-to-vigorous physical activity (MVPA) per week for health [3]. Individuals can meet or exceed the PA guidelines and still spend the majority of the day in sedentary behaviours [4]. The adverse health impacts associated with sedentary behaviours have been shown to occur independently of the amount of PA undertaken [4,5]. A recent review using accelerometer-based estimates indicated that adults spend approximately 8.2 h of their waking day in sedentary behaviours [6]. The 2020 World Health Organization (WHO) guidelines for PA provide specific recommendations to reduce sedentary behaviours at the population level, highlighting the importance of behaviour change interventions to influence the full continuum of PA behaviours [7].

Behaviour change interventions are increasingly used to elicit change in PA [8]. The majority of research has focused on promoting changes in MVPA; studies examining how interventions aiming to increase PA influence accelerometer-measured sedentary behaviours are less prevalent [9,10]. Health outcomes are impacted by the full spectrum of PA behaviours. The potential relationship between PA and sedentary behaviours is relevant, as health risk behaviours have a propensity to co-occur [11]. It is important to understand if a behaviour change intervention that positively impacts PA behaviour can influence sedentary behaviours [12].

A meta-analysis of PA interventions found that interventions designed to elicit PA changes had no significant effect on sedentary behaviours [9]. Prince and colleagues reported that interventions designed for PA change, or interventions designed to target PA and sedentary behaviours together, resulted in small and inconsistent reductions in sedentary behaviours [13]. Given that population health is likely to be improved through increases in PA and reductions in sedentary behaviours [4,5], it is important to understand if PA interventions simultaneously influence time spent sedentary, or if a more concerted approach is required to address sedentary behaviours.

The Healthy 4U-2 (H4U-2) study examined the effectiveness of a PA coaching intervention for changes in MVPA in insufficiently physically active ambulatory hospital patients [14]. Integrated motivational interviewing and cognitive behaviour therapy was used as the theoretical framework for the intervention, with the content designed to increase motivation and self-efficacy for PA change and maintenance. The H4U-2 intervention was designed to change PA and not sedentary behaviours. Understanding the impact that an intervention designed to increase PA has on sedentary behaviours can assist in the design of future behaviour change interventions. Therefore, the aim of this secondary analysis was to examine changes in sedentary behaviours among insufficiently physically active adults recruited into a behaviour change intervention designed to increase PA.

## 2. Materials and Methods

### 2.1. Study Design

The Healthy 4U-2 study was a randomised controlled trial examining the effectiveness of a PA coaching intervention for insufficiently physically active adults recruited from an ambulatory hospital clinic. The intervention was delivered over 3 months, followed by a no-contact maintenance phase up to 9 months [14]. The study was designed and reported in accordance with the (Consolidated Standards of Reporting Trials) CONSORT statement and checklist; the original manuscript contains completed CONSORT and TIDieR checklists [14]. Data were collected at the Bendigo Health Hospital between 2019 and 2020. Ethical approval was granted by the governing hospital and university ethics committees. The trial was prospectively registered with the Australian and New Zealand Clinical Trials Registry (ACTRN12619000036112) prior to participant recruitment.

A full description of the study method was previously published [14]. In brief, we recruited 120 insufficiently physically active adults from ambulatory hospital clinics at a major regional hospital in Victoria, Australia. The primary aim of the H4U-2 study was to increase objectively measured MVPA.

### 2.2. Intervention

All participants completed a 30 min education session prior to group allocation. The education session used self-determination theory [15] to deliver facilitated learning material on determinants of PA self-management and PA behaviour change to support participants around changing PA.

The intervention group undertook a PA coaching intervention that comprised integrated motivational interviewing and cognitive behaviour therapy. Motivational interviewing was used as the foundational approach; the cognitive behaviour therapy strategies were delivered within the motivational interviewing framework to assist PA change. The intervention was directive towards PA change, but in keeping with the spirit and processes of motivational interviewing, participants focused on issues relevant to them and their PA desires and set their own PA goals. The intervention group received 5 × 20 min phone calls over a 12 week period. The intervention was delivered by the first author, a senior physiotherapist. The clinician was trained and experienced in MI-CBT (motivational interviewing and cognitive behaviour therapy) delivery, including participation in a previous MI-CBT intervention study [16]. A detailed description of the intervention content, theory and PA determinants are provided elsewhere [14]. The design of the intervention was based on PA determinants, and participants did not receive any coaching or material directed towards a reduction in sedentary behaviours.

### 2.3. Outcomes

All outcome measures were recorded at baseline, post-intervention (3 months) and follow-up (9 months). Sedentary behaviours were measured with a hip-worn tri-axial accelerometer (wGT3X-BT; Actigraph, USA). Participants wore the accelerometer during all waking hours for 7 consecutive days, excluding sleep and water-based behaviours [17]. Sedentary behaviour was calculated using the ActiLife software (version 6.13.4; ActiGraph, Pensacola, FL, USA) and applying the guidelines by Choi et al. [18]. Non-wear time was defined as 60 min or more of zero counts, with allowance for up to 2 min of observations of <100 counts per minute (CPM) within the non-wear interval. A minimum of 10 h a day was required to qualify as a valid day of measurement. Using the ActiLife software, all valid days of measurement were processed and aggregated to 60 s epochs to permit the application of PA and sedentary cutoff points [19]. Every minute of accelerometer-derived wear time was classified according to the measured intensity (cpm), where a threshold of <100 cpm defined sedentary behaviour [19]. The total time per day spent in sedentary behaviours was determined by summing the minutes in each valid day where the intensity measured below 100 cpm. To calculate sedentary bouts, a minimum period of 10 consecutive minutes where the accelerometer registered <100 cpm with no interruption was used [18,19]. The total number of sedentary bouts represents the number of sedentary bouts detected in the dataset. The daily average of time spent in sedentary bouts was calculated by dividing the total length of sedentary bouts by the total valid days in the dataset. The average length of sedentary bouts was quantified by dividing the total length of sedentary bouts by the total number of sedentary bouts. The proportion of the day spent in sedentary behaviours was attained by dividing the total minutes registered in sedentary behaviours by the total accelerometer wear time.

### 2.4. Analysis

All the analyses were performed using IBM SPSS Statistics for Windows (Version 27.0; IBM Corp., Armonk, NY, USA). Shapiro-Wilk tests were used to assess for normal distribution. The homogeneity of variances was assessed using Levene’s test. Box’s M test was used to assess the homogeneity of covariances. To examine the effect of the PA coaching intervention on all outcomes, mixed-model ANOVAs (within: time; between: intervention) were used. We interpreted a significant interaction effect as indication that the change in outcome was a result of the treatment group allocation. Effect sizes were calculated in IBM SPSS and presented as Partial Eta squared. Mauchly’s test of sphericity was examined, and where the assumption of sphericity was violated, the results were interpreted using a Greenhouse–Geisser correction. Where statistically significant interaction effects were found, analyses of simple main effects were carried out. Where data were found to breach the Shapiro-Wilks test of normality, sensitivity analyses were performed. We inspected the data for significant outliers. The outliers were then removed before performing repeat sensitivity analyses. The result of the sensitivity analyses were consistent with the primary analyses and showed that the outliers did not significantly influence the outcome. As such, all the data were included in the analyses. Analyses were performed using the intention-to-treat principle. Missing data was imputed using the last observation carried forward (LOCF) method [20]. Further sensitivity analyses were carried out, analyzing the data with and without imputing the LOCF value [20]. The repeated sensitivity analyses indicated that the imputation of the LOCF values had no significant impact on the outcomes. As a result, all data were included in analyses.

## 3. Results

**Figure 1:**
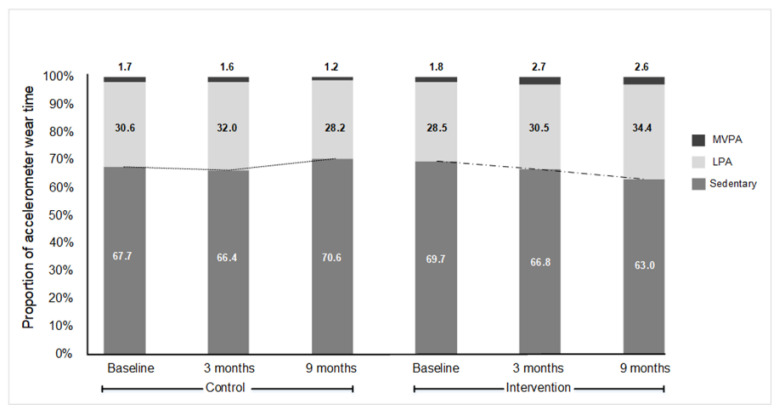
Proportion of accelerometer wear-time in moderate-to-vigorous physical activity (MVPA), light physical activity (LPA) and sedentary behaviours.

A total of 120 participants were enrolled into the study. Baseline outcome measures were completed for all participants prior to group allocation. The attrition rate was low; data was available for 115 participants at 3 months and for 108 participants at 9 months. Participant flow though the study is shown in Supplementary Figure S1. The demographic and clinical characteristics between the groups are shown in Table 1. On average, participants were aged 53 ± 8 years with a BMI (Body mass index) of 31 ± 4 kg/m^2^; 68% identified as female. Participants wore the Actigraph for an average of 14  ±  3 h/day and 6.1  ±  0.8 days/week (out of 7 days/week) at each time-point. At the baseline measurement, the participants spent 553 ± 87 min/day in sedentary behaviours. This equated to the participants spending 69 ± 6% of their day in sedentary behaviours. Accelerometer wear-time indicated that participants spent 29 ± 7% of their day in light physical activity (LPA) and 2 ± 1% in MVPA at baseline (Figure 1).

### Sedentary Behaviour Outcomes

**Figure 2:**
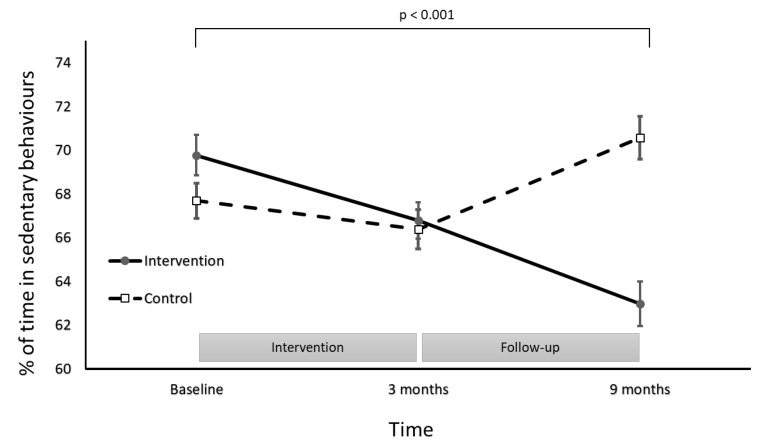
Proportion of time in sedentary behaviours for the intervention and control groups at baseline, post-intervention and follow-up.

Patterns for the groups differed over time (group × time interaction effect: *F*(2236) = 16.98, *p*  <  0.001; Figure 2) for the changes in the proportion of time spent in sedentary behaviours. Participants in the intervention group significantly decreased the proportion of the day spent in sedentary behaviours to at the 9 month follow-up, a decrease of 7% from baseline. In comparison, participants in the control group increased the time spent in sedentary behaviours from baseline to the 9 month follow-up by 3%. The proportion of accelerometer wear-time in MVPA, LPA and sedentary behaviours is detailed in Figure 2.

**Figure 3:**
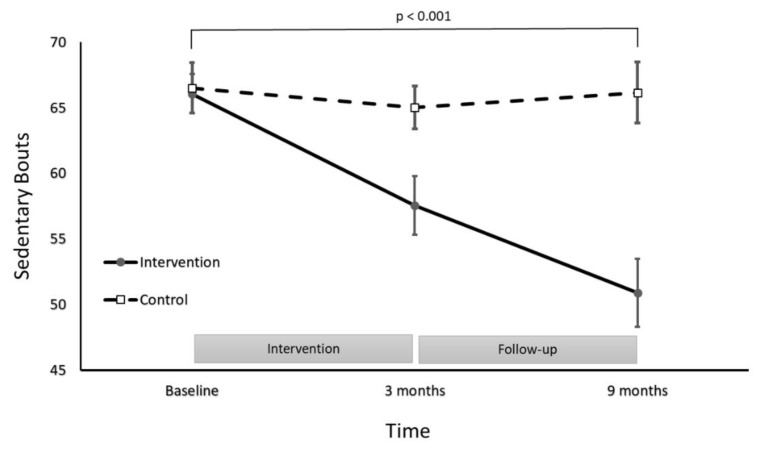
Number of sedentary bouts for the intervention and control groups at baseline, post-intervention and follow-up.

A significant group by time interaction was found for the number of sedentary bouts (*F*(2236) = 7.13, *p*  <  0.001; Figure 3), where the intervention group significantly decreased the total number sedentary bouts at post-intervention by 8 bouts/day (95%CI: 6–10 bouts/day) and by 16 bouts/day (95%CI: 12–20 bouts/day) at follow-up. Relative to baseline, this represented a 24% decrease in the number sedentary bouts at the 9 month assessment. The total number of sedentary bouts remained constant in the control group over the same period, although the differing pattern over time for the intervention group translated to a between-group difference of 16 bouts/day (95%CI: 12–20 bouts/day) at 9 months.

Group by time interactions for daily average of time spent in sedentary bouts and average length of sedentary bouts did not reach significance (Table 2).

## 4. Discussion

The aim of this study was to assess changes in sedentary behaviours in insufficiently physically active ambulatory care patients participating in a 12 week PA coaching intervention. The PA coaching intervention increased objectively measured MVPA, relative to control [14]; given the independent health implications of insufficient PA and sedentary behaviours, it was important to understand what secondary effect the intervention had on sedentary behaviours.

The participants spent the majority of accelerometer-derived wear-time in sedentary behaviours at baseline, engaging in more than 9 h of sedentary behaviours per day. The PA coaching intervention resulted in statistically significant reductions in the total number of sedentary bouts and the overall proportion of time spent in sedentary behaviours. These positive secondary changes were maintained at 9 months. Overall, our results demonstrate that insufficiently physically active ambulatory hospital patients increased MVPA and reduced sedentary behaviours as a result of a PA coaching intervention that was not designed to influence sedentary behaviours. As MVPA and sedentary behaviours are both independent key factors for health [4,5], it is encouraging that the PA coaching intervention decreased overall sedentary time, and the improvements in MVPA were not potentially undermined by an increase in sedentary behaviours.

The PA coaching intervention resulted in significant increases in PA over time, where the intervention group completed 22 min/day (95%CI: 20–25 min/day) of accelerometer-measured MVPA at 9 months. The 7% reduction of sedentary behaviours in the intervention group is not accounted for by this magnitude of change in MVPA. The reductions in sedentary behaviour in the intervention group also resulted in an increase in the amount of the day spent in LPA. This finding is consistent with results presented by Healy et al. (2011), who described a robust inverse correlation between accelerometer-derived LPA and sedentary behaviours (Spearman’s rho = 0.98) [21]. The intervention group increased the proportion of time spent in LPA from 29 ± 7% at baseline to 34 ± 8% at follow-up. This translated to an additional 53 min/day (95%CI: 35–72 min/day) of LPA. The reallocation of sedentary time to PA of any intensity can result in beneficial health outcomes [6,22]. Reallocating 30 minutes of sedentary behaviours to MVPA was found to result in 2–25% improvement in cardiometabolic biomarkers of risk in other studies [6,22]. Swapping 30 sedentary minutes for the equivalent time in LPA improved cardiometabolic risk markers by 2–4% [6]. This highlights that the reductions in sedentary behaviours observed in our study are likely to have important clinical implications, irrespective of whether the sedentary time was replaced with MVPA or LPA.

The efficacy of interventions to influence sedentary behaviours has been shown to vary when the outcomes are reported in proportions or as averages of time [23]. This held true in this study, with significant between-group differences observed for changes in the proportion of time spent sedentary. At the 9 month follow-up period, although the intervention group decreased the daily average of sedentary bouts by 72 min/day (95%CI: 35–106 min/day) the pattern of change did not indicate a significant interaction effect, relative to control. Prince et al. [13] argued that changes in daily percentage of time spent sedentary might be a more meaningful measure of overall sedentary behaviours than daily averages in sedentary behaviours, which adds potential impact to the findings of this study. The decrease in daily percentage of time spent sedentary found in our study was similar to that reported in a multicomponent intervention that specifically aimed to reduce daily sedentary time [23]. Furthermore, the degree of change in sedentary behaviour outcomes aligns with the findings of two systematic reviews and meta-analyses investigating interventions used to decrease sedentary time in adult populations [9,13,24].

Compared to the control, participants who completed the PA coaching intervention significantly decreased the total number of sedentary bouts recorded. The intervention group decreased total sedentary bouts to 50 at the 9 month follow-up, which represented a 24% decrease from baseline. The intervention group did exhibit an increase in the average length of those sedentary bouts over time, though the increase was not statistically significant. Extended, uninterrupted bouts of sedentary time result in worsening cardiometabolic outcomes when compared to sedentary behaviours that are regularly interrupted [25]. A reduction in prolonged sedentary time can go towards mitigating this health risk [7,26]. The PA coaching intervention in this study significantly reduced the number of sedentary bouts without producing a significant compensatory increase in the average duration of sedentary bouts.

Interventions that aimed to increase PA have demonstrated mixed results in eliciting changes in sedentary behaviours. Two meta-analyses indicated that interventions designed with specific strategies to influence sedentary activities are more effective at decreasing sedentary behaviour outcomes when examined against interventions that targeted increasing PA alone or interventions that targeted both PA and sedentary behaviours simultaneously [9,13]. Studies investigating counselling or coaching interventions to increase PA have also demonstrated varied effects on sedentary behaviours [27,28,29]. In a number of studies, unintended increases in sedentary behaviours have been observed as a result of counselling or coaching interventions designed to increase PA [27,28]. Siddique and colleagues found that a PA coaching intervention designed to increase PA also decreased sedentary behaviours [29]. In the same study, participants who received a PA coaching intervention designed to decrease sedentary behaviours did not significantly change sedentary behaviours or PA [29]. Although our intervention did not include specific components relating to sedentary behaviours, the intervention group increased MVPA and decreased sedentary behaviours. This result may have been influenced by the study recruitment and the framework used in the PA coaching intervention.

Participants in our study were recruited based on an interest in being more physically active, though the choice of PA undertaken and the exercise intensity was determined by the individual. The PA coaching used an integrated motivational interviewing and cognitive behaviour therapy framework that was designed to build motivation and self-efficacy for change as well as to facilitate skill development for behaviour change maintenance [30]. This coaching intervention resulted in measured increases in MVPA, though the proportion of time spent in MVPA in the intervention group remained consistent between the 3 month and 9 month follow-up. The increased magnitude of change in the proportion of time spent in sedentary behaviours can be accounted for by the reallocation of sedentary time to LPA. Although the integrated motivational interviewing and cognitive behaviour therapy intervention was directive towards PA change, participants were encouraged to choose their own PA options, including PA of differing intensities. Participants were not required to set rigid goals or specifically tasked to attain the recommended 150 min of MVPA per week. The combination of the participants setting PA goals relevant to them and the coaching support provided to achieve and maintain those goals likely contributed to the overall increase across all PA levels and the resulting decrease in time spent in sedentary behaviours [30]. Future studies should look at the efficacy of integrated motivational interviewing and cognitive behaviour therapy interventions for decreasing sedentary behaviours where this is the primary aim of the intervention or where interventions target changes in sedentary behaviours and PA at the same time.

This study had a number of limitations. The study participants were all enrolled at a single tertiary medical hospital. The participants predominantly identified as female, and the average BMI was 31 ± 4 kg/m^2^. These characteristics might limit the broad generalisability of these findings. Some limitations with hip-worn accelerometers have been observed [31]. The sedentary behaviour cutoff point derived from a single accelerometer axis does not differentiate whether individuals are standing or sitting, which could potentially result in standing still being misclassified as a sedentary activity [31]. The limitations associated with the use of hip-worn accelerometers notwithstanding, accelerometers are established methods for measuring sedentary behaviours, reducing issues such as recall and response biases that limit self-reported measures [32]. Future studies should consider the use of a measurement tool that uses inclinometers, which may offer potential to differentiate between standing and sitting positions [33].

## 5. Conclusions

This study shows that a PA coaching intervention designed to increase MVPA led to a decrease in the proportion of time in sedentary behaviours and the number of sedentary bouts. The findings indicate that the effectiveness of the PA coaching intervention for increasing MVPA was not potentially weakened by compensatory increases in sedentary behaviours. The increased MVPA was instead accompanied by a reduction in sedentary time, with a reallocation of sedentary time into the different categories of PA. The findings of this study would be strengthened by examining the intervention outcomes across different population groups. In addition, an examination of PA and sedentary outcomes following a PA coaching intervention that targets both behaviours is warranted.

## Figures and Tables

**Table 1:** Characteristics of participants at baseline.

Variable	Intervention	Control	*p*-Value
***n***	60	60	
**Age (years)**	54 ± 8	53 ± 7	0.46 ^a^
**Sex: female, *n* (%)**	40 (67%)	41 (68%)	0.84 ^b^
**Stature (cm)**	165 ± 9	167 ± 7	0.17 ^a^
**Weight (kg)**	84.5 ± 9.9	84.3 ± 9.1	0.92 ^a^
**BMI (kg/m^2^)**	31.0 ± 4.4	30.0 ± 4.2	0.19 ^a^
**MVPA (min/day)**	14.7 ± 5.2	14.3 ± 4.7	0.67 ^a^
**Number of sedentary bouts**	66 ± 12	67 ± 15	0.40 ^a^
**Daily average of time spent in sedentary bouts (mins)**	551 ± 77	555 ± 96	0.25 ^a^
**Average length of sedentary bouts (mins)**	60 ± 14	61 ± 16	0.71 ^a^
**Proportion of day in sedentary**	70 ± 7	68 ± 6	0.09 ^a^
**Smoker, *n* (%)**	7 (10%)	5 (10%)	0.71 ^b^
**Obesity, *n* (%)**	30 (50%)	32 (53%)	0.64 ^b^
**Hypertension, *n* (%)**	20 (33%)	18 (30%)	0.66 ^b^
**OA/RA, *n* (%)**	22 (37%)	20 (33%)	0.71 ^b^
**Depression/anxiety, *n* (%)**	12 (20%)	13 (22%)	0.74 ^b^

Group data expressed as means ± standard deviations. Figures in parentheses are proportions. BMI: Body mass index; MVPA: Moderate-to-vigorous physical activity; OA: Osteoarthritis; RA: Rheumatoid arthritis. ^a^ *t*-test between intervention and control groups. ^b^ chi square test between intervention and control groups.

**Table 2:** Changes in sedentary behaviours at all assessment time points.

Outcome		Control			Intervention		Analyses	
	Baseline	3 Months	9 Months	Baseline	3 Months	9 Months	Time x Group (F) ^a^	Effect Size ^b^
**Total sedentary bouts**	67 ± 15	65 ± 13	66 ± 18	66 ± 12	58 ± 17	50 ± 20	7.13 *	0.57
**Daily average of sedentary bouts (min)**	551 ± 77	533 ± 77	538 ± 137	555 ± 98	506 ± 91	472 ± 137	2.93	0.024
**Average length of sedentary bouts (min)**	61 ± 16	59 ± 15	62 ± 32	60 ± 14	69 ± 31	76 ± 40	2.31	0.019
**Proportion of day spent sedentary (%)**	68 ± 6	66 ± 7	71 ± 8	70 ± 7	68 ± 6	63 ± 8	16.98 *	0.126
**MVPA (min/day)** [14]	14 ± 5	13 ± 6	10 ± 6	15 ± 5	23 ± 10	22 ± 10	28.7 *	0.20

Group data are means ± standard deviations. MVPA: moderate-to-vigorous physical activity. * *p*  <  0.05. ^a^ Interaction effect of time by group on dependent variable. ^b^ Partial eta-squared. [14]

## Data Availability

The data presented in this study are available on reasonable request from the corresponding author.

## Supplementary Materials

The following are available online at https://www.mdpi.com/article/10.3390/ijerph18115543/s1, Figure S1: Flow of Study.
