# Supplementary figures and images for: The Effect of a Physical Activity Coaching Intervention on Accelerometer-Measured Sedentary Behaviours in Insufficiently Physically Active Ambulatory Hospital Patients
Source: Int J Environ Res Public Health. 2021 May 22;18(11):5543. doi: 10.3390/ijerph18115543 (PMC8196832; doi:10.3390/ijerph18115543)

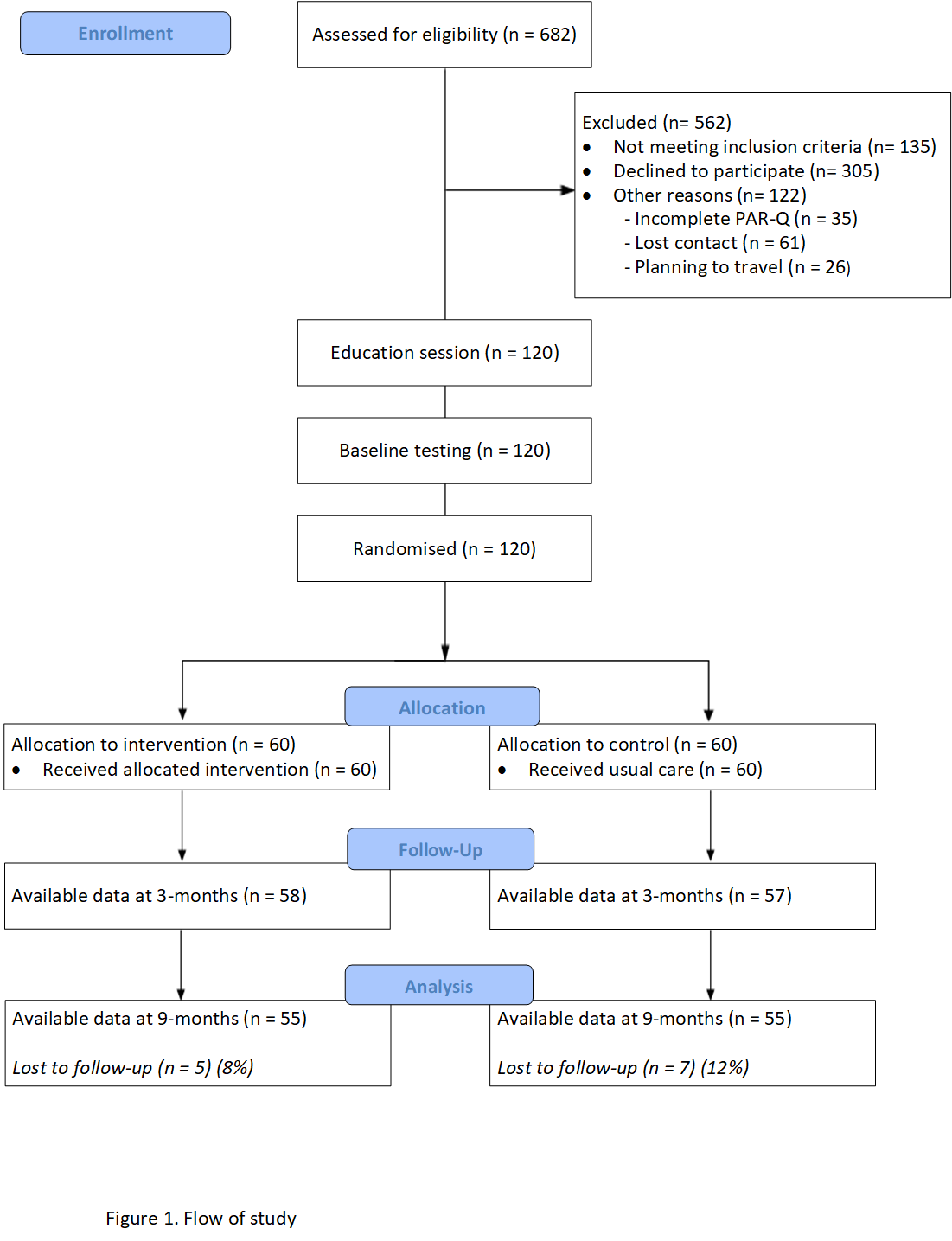

Supplement: Supplementary file 1 [file ijerph-18-05543-s001.zip › ijerph-1181380-supplementary.tif]
